# Epistasis between *IL1A*, *IL1B*, *TNF*, *HTR2A*, *5-HTTLPR* and *TPH2* Variations Does Not Impact Alcohol Dependence Disorder Features

**DOI:** 10.3390/ijerph6071980

**Published:** 2009-07-16

**Authors:** Antonio Drago, Ioannis Liappas, Carmine Petio, Diego Albani, Gianluigi Forloni, Petros Malitas, Christina Piperi, Antonis Politis, Elias O. Tzavellas, Katerina K. Zisaki, Francesca Prato, Sara Batelli, Letizia Polito, Diana De Ronchi, Thomas Paparrigopoulos, Anastasios Kalofoutis, Alessandro Serretti

**Affiliations:** 1 Institute of Psychiatry, University of Bologna, Italy; E-Mails: antonio.drago@unibo.it (A.D.); diana.deronchi@unibo.it (D.D.R.); 2 Department of Psychiatry, Eginition Hospital, University of Athens Medical School, Athens, Greece; E-Mail: iliappas@eginitio.uoa.gr; 3 Neuroscience Department. Istituto di ricerche Farmacologiche “Mario Negri”, Milan, Italy; E-Mails: albani@marionegri.it (D.A.); forloni@marionegri.it (G.F.); 4 European Centre for the Quality of Life – E.C.Qua.L., Athens, Greece; E-Mail: pnmalitas@ecqual.org; 5 Laboratory of Biological Chemistry University of Athens Medical School, Athens, Greece; E-Mail: cpiperi@med.uoa.gr; 6 Division of Geriatric Psychiatry Department of Psychiatry, Eginition Hospital, University of Athens Medical School, Athens, Greece; E-Mail: apolitis@med.uoa.gr; 7 Department of Pharmacology, University of Athens Medical School, Athens, Greece; E-Mail: kzisaki@ecqual.org; 8 Biomedical Research Foundation of Academy of Athens, Greece; E-Mail: akalofoutis@med.uoa.gr; 9 Department of Mental Health, Azienda USL di Bologna, Bologna, Italy; E-Mail: carmine.petio@ausl.bologna.it

**Keywords:** gene, alcohol dependence, IL1A, IL1B, TNF, 5HTPR, HTR2A, TPH2, association

## Abstract

We assessed a set of biological (HDL, LDL, SGOT, SGPT, GGT, HTc, Hb and T levels) and psychometric variables (investigated through HAM-D, HAM-A, GAS, Liebowitz Social Anxiety Scale, Mark & Mathews Scale, Leyton scale, and Pilowski scale) in a sample of 64 alcohol dependent patients, at baseline and after a detoxification treatment. Moreover, we recruited 47 non-consanguineous relatives who did not suffer alcohol related disorders and underwent the same tests. In both groups we genotyped 11 genetic variations (rs1800587; rs3087258; rs1799724; *5-HTTLPR*; rs1386493; rs1386494; rs1487275; rs1843809; rs4570625; rs2129575; rs6313) located in genes whose impact on alcohol related behaviors and disorders has been hypothesized (*IL1A*, *IL1B*, *TNF*, *5-HTTLPR*, *TPH2* and *HTR2A*). We analyzed the epistasis of these genetic variations upon the biological and psychological dimensions in the cases and their relatives. Further on, we analyzed the effects of the combined genetic variations on the short – term detoxification treatment efficacy. Finally, being the only not yet investigated variation within this sample, we analyzed the impact of the rs6313 alone on baseline assessment and treatment efficacy. We detected the following results: the couple rs6313 + rs2129575 affected the Leyton -Trait at admission (p = 0.01) (obsessive-compulsive trait), whilst rs1800587 + *5-HTTLPR* impacted the Pilowski test at admission (p = 0.01) (hypochondriac symptoms). These results did not survive Bonferroni correction (p ≤ 0.004). This lack of association may depend on the incomplete gene coverage or on the small sample size which limited the power of the study. On the other hand, it may reflect a substantial absence of relevance of the genotype variants toward the alcohol related investigated dimensions. Nonetheless, the marginal significance we detected could witness an informative correlation worth investigating in larger samples.

## 1. Introduction

Genes coding for: interleukin 1, alpha (*IL1A*), interleukin 1, beta (*IL1B*), tumor necrosis factor (*TNF*), serotonin receptor 2A (*HTR2A*), the promoter of the serotonin transporter gene (*5-HTTLPR*) and tryptophan hydroxylase 2 (*TPH2*) are candidate genes whose deregulation is thought to be central to psychiatric disorders [1]. In particular, the relevance of the serotonin system has been widely investigated and supported in psychopathology [2,3], and related to the molecular disruptions associated with alcohol related disorders [4,5]. Consistently, the genetic variations located in the promoter of the serotonin transporter has been convincingly found to regulate mood disorders [6] and alcohol related disorders [7–9]. Conflicting results [10,11] have further stimulated research in this direction. *HTR2A* plays as a key serotonin receptor: it is responsible for the post-synaptic activation upon serotonin transmission, a role that may modulate the psychiatric-related neuronal deregulations [12]. Recent evidence supports its connection with alcohol addiction [13–15]. In particular, *HTR2A* A-1438G variation, which is in completely LD with the variation we investigated here (reviewed in [12]), was found to be associated with alcohol dependence [15] and tobacco smoking combined or not with alcohol dependence [13]. Furthermore, *HTR2A* rs6313, which regulates the expression rate of *HTR2A* (reviewed in [12]), was found to be associated with alcohol abuse in male patients [14]. *TPH2* is the brain specific rate limiting enzyme in the synthesis of serotonin [16], a role that candidates it as a modulator of the serotonin system orchestration. Recent reports discouraged the hypothesis of its participation to alcohol related behaviors [17–19]. *IL1A* and *IL1B* are mediators of the acute phase response, some genetic variations located in these genes have been associated with depressive symptomatology, antidepressant outcome and alcoholism [20,21]. *TNF* is a white cell produced pro-inflammatory cytokine likely involved in depressive disorder [16] which has been poorly investigated as a mediator of alcohol related psychiatric disorders. We yet investigated a set of variations located in the *IL1A* (rs1800587), *IL1B* (rs3087258), *TNF* (rs1799724), *5-HTTLPR* (*5-HTTLPR*) and *TPH2* (rs1386493; rs1386494; rs1487275; rs1843809; rs4570625; rs2129575) as modulators of alcohol related disruptions, both enzymatic and psychometric in previous published papers [22,23]: despite the relevance of these receptors and enzymes in the modulation of alcohol related phenotypes, we reported a lack of association. The reasons of this may be attributable to: lack of power, incomplete coverage of genetic mutations, incomplete analysis of epigenetic events, incomplete analysis of socio-demographic events and so on. In particular, epistasis between gene may have been played a fundamental role which remained undiscovered during the analysis of single mutations in previous papers we published on this sample. The aim of this paper is to challenge this particular limit of the previous publications on this sample [6,22] through the analysis of the epistasis between the set of variations yet singularly analyzed, also including a not yet investigated mutation (*HTR2A* rs6313) in the model. Further on, given that *HTR2A* rs6313 was not investigated as a single mutation in this sample of patients, we investigated the *HTR2A* rs6313 alone impact as a regulator of the biological and psychometric alcohol related variables.

## 2. Methods

### 2.1. Inclusion and Exclusion Criteria

To be included in the study patients had to fulfill the DSM-IV (Diagnostic and Statistical Manual of mental disorders) diagnostic criteria for alcohol abuse/dependence – “primary alcoholism” – they had to have no recent alcohol intake (average period of abstinence prior to the admission to the clinic = 24.0 ± 12.2 h), and to give full voluntary consent to the study. Other inclusion criteria were: absence of serious physical illness (as assessed through physical examination and routine laboratory screening), absence of other drug abuse, age between 20 and 75 years old, absence of DSM-IV axis I co-morbidity. The presence of affective symptoms was not considered to be an exclusion criterion. Alcohol abusers who fulfilled the DSM-IV diagnosis of depressive disorder were excluded from the study in the case that a full diagnosed depressive episode was precedent to the onset of the alcohol dependence. Relatives (non-consanguineous) were included in the study if they did not meet criteria for past or current alcohol abuse/dependence and major medical disturbances. Written informed consent was obtained from each participant after approval from the local ethical committee. Patients and their relatives were enrolled at the Drug and Alcohol Addiction Clinic of the Athens University Psychiatric Clinic at the Eginition Hospital in Athens, Greece. For patients, alcohol detoxification program included vitamin replacement (B, C, E) and oral administration of diazepam (10–40 mg daily in divided doses), with gradual taper off over a week. The overall period of detoxification was completed in 4–5 weeks. Patients then followed an inpatient standard treatment program with a short-term psychotherapy of cognitive-behavioral orientation.

### 2.2. Assessment

The biochemical profile of cholesterol, high density lipoprotein (HDL), low density lipoprotein (LDL), glutamic oxaloacetic transaminase (SGOT or AST), serum glutamic pyruvic transaminase (SGPT or ALT) and gamma-glutamyltransferase (GGT), were assessed once in relatives and twice in patients. Hematocrit, hemoglobin and thyroid hormonal levels (Triiodothyronine = T3, Thyroxine = T4, Thyrotropin = TSH) were measured once in both relatives and alcohol dependents. Patients were evaluated by a set of psychological tests: at admission they were evaluated by the Schedules for Clinical Assessment in Neuropsychiatry (SCAN [24]) and the Composite International Diagnostic Interview (CIDI - section on alcohol consumption [25]). Sociodemographic data were collected as well. Hamilton Depression Rating Scale (HAM-D) the Hamilton Anxiety Rating Scale (HAM-A) the Global Assessment Scale (GAS), the Liebowitz Social Anxiety Scale (social fear and avoidance) [26], the Mark & Mathews Scale (general fear) [27], the Leyton Obsessional Inventory [28], and the Pilowski scale (hypochondriac symptoms) [29] were administered at admission and discharge. Genotyping was performed as previously described [22].

### 2.3. Statistics

#### Principal analysis

In the principal analysis the independent variable was the epistasis between the investigated variations (rs1800587; rs3087258; rs1799724; *5-HTTLPR*; rs1386493; rs1386494; rs1487275; rs1843809; rs4570625; rs2129575; rs6313). Diagnosis, biological and psychometric tests’ scores were treated as dependent variables of the principal analysis. Correlation analysis was employed in order to assess whether socio-demographic variables were associated with the dependent variables. In the event of significant association, socio-demographic variables were treated as covariates in the principal analysis. We employed GMDR Software Beta version 0.7 to investigate the gene to gene interactions [30], possible confounders were included in the analysis accordingly to GMDR manual [30]. Gender and age were included as possible confounders when analyzing cases versus controls. Alpha value was conservatively set to 0.004 (Bonferroni correction: 0.05/11 polymorphisms). Power was sufficient in our sample to detect an effect size of d = 0.88 or similar as previously detailed [22]. The multifactor dimensionality reduction (MDR) method [30] was employed by the GMDR Software Beta version 0.7 to investigate the gene–gene interactions. The MDR model computes the data reducing the n-dimensional space formed by a given set to a single dimension to analyze n-way interactions. Each class is then classified as either high risk or low risk according to the relative proportion of cases to controls in that class. This is performed for all possible combinations of SNPs, and the combination with the best predictive capacity is identified. Accordingly to the settings of the software, we tested all the possible two- to six-loci interactions using 10-fold cross-validation in an exhaustive search, thus considering all possible SNP combinations. As outcome parameters we considered the testing balanced accuracy, the significance test, along with the test sensitivity and specificity. The testing balanced accuracy measures the degree to which the interaction accurately predicts case–control status (label 1 indicating good prediction of the model, label 0.50 suggesting that the model is no better than chance in selecting cases from controls). The significance test gives the p value of the calculation.

#### Secondary analysis

In the secondary analysis the independent variable was: rs6313 genotypes. Diagnosis, biological and psychometric tests’ scores were treated as dependent variables. Chi-square test, ANOVA and ANCOVA analyses were employed when needed in order to infer the impact of rs6313 genotypes on the dependent variables (diagnosis, biological and psychometric tests’ scores).

## 3. Results

Sixty four alcohol dependent patients and 47 relatives (non-consanguineous) were included in the study. Patients’ and their relatives’ characteristics are reported in Table 1. Table 2 reports the psychological assessment at admission and discharge. Biochemical reports are detailed in [22]. As a result of the principal analysis, that is, the interaction between epistasis (independent variable) and diagnosis and psychometric tests’ scores (dependent variable), we found a marginal impact of *HTR2A* rs6313 + *TPH2* rs2129575 on Leyton -Trait at admission (p = 0.01; test accuracy = 0.87; test sensitivity = 0.95; test specificity = 0.56) and a similar marginal impact of ILA rs1800587 + *5-HTTLPR* on Pilowski test at admission (p = 0.01; test accuracy = 0.80; test sensitivity = 0.73; test specificity = 0.85).

**Figure 1.**
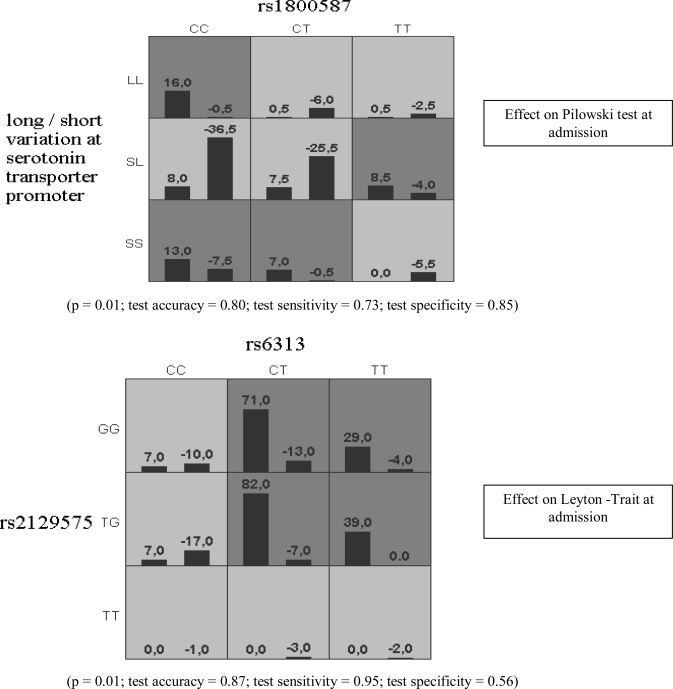
Marginal significant epistasis results. **Dark gray cells** = the combinations for which the combined frequencies of genotypes can significantly predict the distribution of the dependent variable. **Light gray cells** = the combinations that cannot predict the distributions of the dependent variable. **Bars in each box** = the frequency of the combined genotypes are separated on the basis of the dependent variable into two groups, being the mean score of the dependent variable the threshold between them. **Numbers upon each column** = given that the dependent variable was implemented as a normalized distribution with “0” as mean and threshold value, columns on the left in each box are labeled as positive, and columns on the right of each box are labeled as negative. Absolute numbers upon each column represent the frequency of each combined genotype.

The results are shown in Figure 1: the frequencies of combined genotypes which have been found to be significantly associated with the scores of the dependent variables are in grey boxes; positive scores upon the right column in every box represent the frequencies of combined genotypes that have been associated with higher scores at the investigated test, negative scores upon each left column in each box represent the frequencies of the combined genotypes which have been associated with lower scores at the investigated test. Higher and lower scores are defined accordingly to the normal distribution obtained from the analysis of input data, “0” corresponding to the mean value of dependent variable, positive scores corresponding to scores upon the mean and negative scores corresponding to scores below the mean in the sample. Light boxes represent not significant associations. These results did not survive the Bonferroni correction. In the secondary analysis of the study, rs6313 did not impact the psychopathologic dimension and did not separate patients from their relatives (data not shown). In the pictures the grey shadowed boxes designate the combinations of genotypes associated with a statistically significant different distribution of the scores of the test under investigation. The light shadowed boxes designate the combinations of genotypes associated with a non statistically significant different distribution of the scores of the test under investigation.

To be implemented in the GMDR software the scores were normalized with “0” as the mean and threshold value. The GMDR software computes the frequencies of subjects who score higher (positive values on the left column on each box) or lower (negative values on the right column on each box) than “0” which is representative of the mean of the scores at the test under investigation. The scores are differentiated in positive and negative: the mean score given as a threshold, the positive scores stand for subjects who ranked higher than the mean, the negative stand for subjects who ranked lower than the mean. The difference in the frequencies of positive and negative scoring subjects is labeled as significant in the grey shadowed boxes. Light shadowed boxes stand for not significant difference in distribution of positive and negative scores. For example, in the first picture it is shown that *IL1A* rs1800587 and *5-HTTLPR* combined variations are associated with a significant different score at Pilowski test at admission only in some specific combination of genotypes: the genotype CC at rs1800587 is associated with higher frequencies of subjects who ranked higher than the mean of the Pilowski test, only when it is found to be in combination with the LL or SS genotypes at the *5-HTTLPR*. On the other hand, the combination with heterozygosis at the *5-HTTLPR* with the same genotype at rs1800587 resulted in a not significant different distribution of subjects who ranked higher or lower than the mean of the Pilowski test. In the second picture, the combination of the CT genotype at rs6313 and the GG genotype at rs2129575 results into statistically significant higher scores at the Leyton – Trait test at admission, along with the other combinations of genotypes which are grey shadowed.

## 4. Discussion

We found a marginal impact of the epistatic effect between the investigated variations and the obsessive-compulsive traits and hypochondriac symptoms. The low level of significance which did not stand the Bonferroni correction, is likely mainly related to the small sample size we investigated here: as formerly reported, the power was sufficient to detect a large effect size. Moreover, the genetic analysis we performed here did not cover the genes that are under investigation, which results in a possible genetic bias: some variations remain hidden in the present investigation that could impact the functionality of the investigated mutations. This exposes our study to a biological stratification bias which is quite common in literature [31]. Finally, the most part of the investigated variations belong to the *TPH2* gene which has been quite consistently reported to be not associated with alcohol dependence. In all facts, even though *TPH2* is the rate limiting enzyme for the synthesis of serotonin in central nervous system [32], a molecular role that candidates it as a central regulator of psychiatric disorders such as major depressive disorder [33–35] and bipolar disorder [35–37], recent reports rejected the hypothesis that variations located in the *TPH2* gene could modulate alcohol dependence [17–19]. This is in contrast with the inhibitory role played by the serotonin system toward the mesolimbic dopaminergic system [38,39] which is thought to be primarily involved in the disruptions that lead to alcohol dependence [5]. This is the biological evidence that steered our attention on the possible epistasis impact of some relevant and well replicated variants (*5-HTTLPR* and *HTR2A* rs6313) over a putative good candidate as the *TPH2* is. Some recent reports on the significant epistasis interaction between genetic variables that did not cast informative results when singularly investigated [40,41], convinced us to re-analyze our previously published results. Unfortunately, we could only find not conclusive data in this specific case. The question arises as whether the marginal significant associations we report are false positive findings, which are a common concern in genetic association studies [42], or the first partial evidence of a significant association.

## Figures and Tables

**Table 1.** Demographic data of patients.

**Variable**	**Total**	**Females**	**Males**

**Sex**	Cases = 64 (100%)	Cases = 15 (21%)	Cases = 49 (79%)
	Controls = 47 (100%)	Controls = 39 (52%)	Controls = 8 (48%)
**Age (years)**	Cases = 45.60 ± 9.89	Cases = 45.42 ± 9.96	Cases = 45.64 ± 9.94
	Controls = 47 ± 11.52	Controls = 48.79 ± 10.98	Controls = 43.87 ± 13.96
**Weight (kg)**	74.42 ± 11.45	62.94 ± 0.96	77.11 ± 9.83
**Alcohol (gr/day)**	265.27 ± 138.32	152.32 ± 55.66	291.77 ± 138.7
**Age of onset**	26.91 ± 10.14	29 ± 8.75	26.42 ± 10.45
**Smoking (cigs/day)**	11.40 ± 15.84	9.74 ± 10.47	11.79 ± 16.89

**Table 2.** Psychopathological assessment: admission and discharge.

**Variable**	**Admission**	**2nd week**	**3rd week**	**4th week**	**P (adm vs 4th week)**

**HDRS**	39.45 ± 6.86	31.35 ± 7.11	14.5 ± 6.81	6.65 ± 5.20	< 0.001
**HARS**	34.44 ± 9.80	27.54 ± 8.47	14.35 ± 7.11	6.71 ± 5.29	< 0.001
**GAS**	46.30 ± 5.06	56.7 ± 5.51	74.3 ± 8.68	83.90 ± 8.27	< 0.001
**Liebowitz Social Anxiety Scale (social fear)**	51 ± 9.45	–	–	30.16 ± 6.04	< 0.001
**Liebowitz Social Anxiety Scale (social avoidance)**	54.91 ± 11.04	–	–	31.89 ± 7.63	< 0.001
**Mark & Mathews Scale**	49.30 ± 11.59	–	–	18.86 ± 5.91	< 0.001
**Leyton Obsessional Inventory**	14.5 ± 3.34	–	–	10.04 ± 3.17	< 0.001
**Pilowski scale**	9.14 ± 2.72	–	–	4.92 ± 2.16	< 0.001

## Abbreviations:

5-HTTLPR: promoter of the gene of the serotonin transporter;
CIDI: Composite International Diagnostic Interview;
GAS: Global Assessment Scale;
GGT: gamma-glutamyltransferase;
GMDR: general multifactor dimensionality reduction;
HAM-A: Hamilton Anxiety Rating Scale;
HAM-D: Hamilton Depression Rating Scale;
Hb: hemoglobin;
HDL: high density lipoprotein;
HTc: hematocrit;
HTR2A: serotonin receptor 2A;
IL1A: interleukin 1, alpha;
IL1B: interleukin 1, beta;
LDL: low density lipoprotein;
MDR: multifactor dimensionality reduction;
SCAN: Schedules for Clinical Assessment in Neuropsychiatry;
**SGOT** (or **AST**): glutamic oxaloacetic transaminase;
**SGPT** (or **ALT**): glutamic pyruvic transaminase;
T levels: thyroid hormonal levels;
T3: Triiodothyronine;
T4: Thyroxine;
TNF: tumor necrosis factor;
TPH2: tryptophan hydroxylase 2;
TSH: Thyrotropin.
